# Universal DNA Methylation Age Across Mammalian Tissues

**DOI:** 10.1093/geroni/igab046.1588

**Published:** 2021-12-17

**Authors:** Viviana Perez Ake Lu, Zhe Fei, Ken Raj, Steve Horvath

**Affiliations:** 1 UCLA, Los Angeles, California, United States; 3 Centre for Radiation, Chemical and Environmental Hazards, Public Health England, Chitlon, England, United Kingdom

## Abstract

Aging is often perceived as a degenerative process caused by random accrual of cellular damage over time. In spite of this, age can be accurately estimated by epigenetic clocks based on DNA methylation profiles from almost any tissue of the body. Since such pan-tissue epigenetic clocks have been successfully developed for several different species, it is difficult to ignore the likelihood that a defined and shared mechanism instead, underlies the aging process. To address this, we generated over 10,000 methylation arrays, each profiling up to 37,000 cytosines in highly-conserved stretches of DNA, from over 59 tissue-types derived from 128 mammalian species. From these, we identified and characterized specific cytosines, whose methylation levels change with age across mammalian species. Genes associated with these cytosines are greatly enriched in mammalian developmental processes and implicated in age-associated diseases. From the methylation profiles of these age-related cytosines, we successfully constructed three highly accurate universal mammalian clocks for eutherians, and one universal clock for marsupials. The universal clocks for eutherians are similarly accurate for estimating ages (r>0.96) of any mammalian species and tissue with a single mathematical formula. Collectively, these new observations support the notion that aging is indeed evolutionarily conserved and coupled to developmental processes across all mammalian species - a notion that was long-debated without the benefit of this new and compelling evidence.

